# Roles of TLR7 in *Schistosoma japonicum* Infection-Induced Hepatic Pathological Changes in C57BL/6 Mice

**DOI:** 10.3389/fcimb.2021.754299

**Published:** 2021-10-08

**Authors:** Yuanfa Feng, Hongyan Xie, Feihu Shi, Dianhui Chen, Anqi Xie, Jiajie Li, Chao Fang, Haixia Wei, He Huang, Xingfei Pan, Xiaoping Tang, Jun Huang

**Affiliations:** ^1^ Department of Infectious Diseases, the Third Affiliated Hospital of Guangzhou Medical University, Guangzhou, China; ^2^ China Sino-French Hoffmann Institute, Guangzhou Medical University, Guangzhou, China; ^3^ Guangzhou Eighth People’s Hospital, Guangzhou Medical University, Guangzhou, China

**Keywords:** TLR7, *S. japonicum*, hepatic inflammation, Th2, macrophages

## Abstract

*S. japonicum* infection can induce granulomatous inflammation in the liver of the host. Granulomatous inflammation limits the spread of infection and plays a role in host protection. Toll-like receptor 7 (TLR7) is an endosomal TLR that recognizes single-stranded RNA (ssRNA). In this study, the role of TLR7 in *S. japonicum* infection-induced hepatitis was investigated in both normal and TLR7 knockout (KO) C57BL/6 mice. The results indicated that TLR7 KO could aggravate *S. japonicum* infection-induced damage in the body, with less granuloma formation in the tissue, lower WBCs in blood, and decreased ALT and AST in the serum. Then, the expression of TLR7 was detected in isolated hepatic lymphocytes. The results indicated that the percentage of TLR7^+^ cells was increased in the infected mice. Hepatic macrophages, DCs, and B cells could express TLR7, and most of the TLR7-expressing cells in the liver of infected mice were macrophages. The percentage of TLR7-expressing macrophages was also increased after infection. Moreover, macrophages, T cells, and B cells showed significant changes in the counts, activation-associated molecule expression, and cytokine secretion between *S. japonicum*-infected WT and TLR7 KO mice. Altogether, this study indicated that TLR7 could delay the progression of *S. japonicum* infection-induced hepatitis mainly through macrophages. DCs, B cells, and T cells were involved in the TLR7-mediated immune response.

## Introduction

Schistosomiasis is the most important helminth disease in the world from a public health perspective (Llanwarne and Helmby, 2021). It is estimated that approximately 230 million people in the world are still under the threat of schistosomiasis (Lackey and Horrall, 2020). *Schistosoma mansoni* and *Schistosoma japonicum* (*S*. *japonicum*) account for the majority of global intestinal schistosomiasis cases, and *S*. *japonicum* is prevalent in East Asia (Wang et al., 2018).

During infection with *S. japonicum*, cercariae, juvenile worms, adult worms and eggs can cause damage to the host (Llanwarne and Helmby, 2021). Eggs are deposited in the liver and intestinal wall of the host and can secrete soluble egg antigen (SEA) (De Marco et al., 2019) and induce egg granuloma formation and liver fibrosis (Schwartz and Fallon, 2018). Granulomatous inflammation can lead to pathological changes, but granulomatous inflammation limits the spread of infection and plays a role in host protection (Hams et al., 2013). Extensive granulomatous fibrosis causes portal hypertension, which results in irreversible cirrhosis and advanced schistosomiasis, leading to host death (Lackey and Horrall, 2020).

The liver microenvironment is a site of immune regulation and tolerance induction, with a unique constituency of innate and adaptive immune cells (Williamson et al., 2019). Liver resident antigen presenting cells (APCs) that have been shown to regulate inflammatory and T cell-mediated immune responses include dendritic cells (DCs), macrophages, sinusoid-lining endothelial cells, and hepatic stellate cells (Racanelli and Rehermann, 2006). Liver-resident macrophages, also called Kupffer cells, account for approximately 90% of the total tissue macrophages in the body. Recent studies have shown that the liver is a primary surveillance organ for intravascular infections and is especially important for filtering pathogens *via* KCs to maintain blood sterility (Knolle and Wohlleber, 2016; Wohlleber and Knolle, 2016).

*S. japonicum* infection can induce a class Th2 immune response in both humans and animals (Farwa et al., 2018). It was reported that many kinds of immune cells, such as DCs, Th cells, B cells, and γδ T cells, take part in the course of the inflammatory response (Kumar et al., 2019; Tang et al., 2019; Xiao et al., 2020). Many kinds of cytokines, including IL-4, IL-10, IL-13, IL-17, and TGF-beta, play important roles in mediating these immune responses (Kassa et al., 2019; Osada et al., 2019; Huwait et al., 2021). In addition, pattern recognition receptors (PRRs), such as Toll-like receptors (TLRs) and mannose receptors (CD206), which recognize pathogen-associated molecular patterns (PAMPs) on *S. japonicum*, were reported to modulate the immune response (Paveley et al., 2011; Wang et al., 2017).

TLR7 is an endosomal TLR that recognizes single-stranded RNA (ssRNA) and responds to imidazoquinoline compounds such as imiquimod or resiquimod (Ernst et al., 2020). TLR7 was originally identified as a sensor for single stranded RNA (ssRNA) and plays an important role in fighting against pathogens, such as viruses and bacteria (Parra et al., 2018; Heni et al., 2020). In parasite research, TLR7 ccould mediate early innate immune responses to malaria (Baccarella et al., 2013). Moreover, TLR7 was reported triggering in neutrophils regulated early innate functions with major consequences on subsequent disease evolution in Cutaneous Leishmaniasis (Regli et al., 2020). Recently, TLR7/8 agonists were found to be therapeutic agents against bacteria (Saroa et al., 2019) and virus infection (Boni et al., 2018). In addition, TLR7/8 agonists were identified as effective candidate adjuvants in vaccine research (Hu et al., 2020). Moreover, TLR7 was shown to be protective in atherosclerosis (Karadimou et al., 2017). The TLR7 agonist R848 was reported to promote survival in cancer (Michaelis et al., 2019).

Here, the role of TLR7 in *S. japonicum* infection was investigated in the livers of C57BL/6 mice, and the mechanism was explored.

## Materials and Methods

### Mice, Parasites, and Infection

Female C57BL/6 mice were purchased from the Animal Experimental Center of Guangzhou University of Chinese Medicine (Guangzhou, China), and TLR7^-/-^ mice (B6.129S1-Tlr7^tm1Fl^v/J, strains: 008380) were purchased from the Jackson Laboratory (Bar Harbor, USA). All mice were maintained under specific pathogen-free conditions and used at 6–8 weeks of age.

*S. japonicum* cercariae were shed from naturally infected *Oncomelania hupensis* snails, which were purchased from Jiangsu Institute of Parasitic Disease (Wuxi, China). The mice were infected as previous reported (Cha et al., 2020). The snails containing *S. japonicum* cercariae were placed in dechlorination water at room temperature and placed in a light environment for about 1h. After the activity and quantity of cercariae met the experimental requirements, a sterile loop was used to transfer the *S. japonicum* cercariaecontaining water on a piece of clean cover slide, and the number of cercariae was counted under a microscope. The abdominal skin of mice was prepared by shaving, and then the cover slide with 40 ± 5 *S. japonicum* cercariae was put on the abdominal skin in close contact for 10 min.

After the whole infection process was completed, mice in the infected group and control group were fed in the Experimental Animal Center of Guangzhou Medical University. Animal experiments were performed in strict accordance with the regulations for the Administration of Affairs Concerning Experimental Animals (S2020-055), and all efforts were made to minimize suffering.

### Reagents and Antibodies

RPMI 1640, FBS, penicillin, and streptomycin were obtained from Invitrogen (Grand Island, NY). The liver dissociation kit, recombinant murine was from Miltenyi Biotec. Phorbol 12-myristate 13-acetate (PMA), brefeldin A, ionomycin, CD3, CD28, and dimethyl sulfoxide (DMSO) were purchased from Sigma-Aldrich (St. Louis, MO). We obtained the following fluorescein-conjugated anti-mouse antibodies from eBioscience (San Diego, CA), Biolegend (San Diego, CA) and BD: CD3e-APC-Cy7 (145-2C11), CD4-PerCP-Cy5.5 (RM4-5), CD8a-PE (53-6.7), ICOS-PE-Cy7 (C398.4A), CD69-BV421 (MIH5), CD19- PE-Cy5(6D5), CD138-PE-Cy7 (281-2), B220-APC-Cy7 (RA3632), CD80-PE (16-10A1), CD86-APC (GL1), CD3e-FITC (145-2C1), CD11b- PE-Cy7 (M1/70), Ly-6C-PerCP-Cy5.5 (HK1.4), F4/80- APC-Cy7 (3M8), CD192/CCR2-BV421 (SA203G11), CX3CR1-APC (SA011F11), CD135-BV421 (A2F10.1), CD11c- PerCP-Cy5.5 (HL3), Gr-1-FITC (RB6-8C5), Ly-6G- APC-Cy7 (1A8), CD287/TLR7-PE (A94B10), CD103-PE (M290), IFN-γ-APC (XMG1.2), IL-4-PE (11B11), IL-2-PE (JES6-5H4), IL-6-APC (MP5-20F3), IL-10-PE (JES5-16E3), IL-13-eFlour450 (ebio13A), IL-17-PE (TC11-18H10.1)), IL-21-APC (FFA21) and their corresponding isotype controls.

### Histology Studies

Parts of the livers were cut and perfused three times with 0.01 M phosphate-buffered saline (pH = 7.4), fixed in 10% formalin, embedded in paraffin, and sectioned. The slices were stained by standard haematoxylin-eosin (H&E) staining and examined by light microscopy under 100× magnification.

### Biochemical Assays

Serum levels of ALT and AST were tested using biochemical kits (Tellgen Life Technology, Shanghai, China), and detected by Beckman Coulter AU5800 (California. USA).

### Isolation of Immune Cells

Mice were sacrificed, and the livers were digested with a LIVER dissociation kit (Miltenyi Biotec, Germany) and dissociated into a cell suspension. Then, immune cells were isolated by Ficoll-Hypaque (DAKEWE, SZ, China) density gradient centrifugation from the cell solution. Isolated cells were washed twice in HBSS and resuspended at 2 × 106 cells/ml in complete RPMI 1640 medium supplemented with 10% heat-inactivated foetal calf serum (FCS), 100 U/ml penicillin, 100 µg/ml streptomycin, 2 mM glutamine, and 50 µM 2-mercaptoethanol.

### RNA Preparation for Real-Time PCR

Lymphocytes were generated following previously described procedures. Total RNA was isolated from the liver immune cells of infected and naive mice using TRIzol Reagent (Invitrogen Life Technologies, Carlsbad, CA, USA) following the manufacturer’s instructions. cDNA was synthesized with HiScript^®^III RT SuperMix for qPCR (+gDNA wiper) (Vazyme Biotech, China), and mRNA expression was determined with ChamQ Universal SYBR qPCR Master Mix (Vazyme Biotech, China) according to the manufacturer’s instructions. The TLR7 primers were synthesized from Invitrogen (Shanghai, China) as follows: 5′-CCA CAT TCA CTC TCT TCA TTG G-3′ (forward) and 5′-GGT CAA GAA CTT CCA GCC TG-3′ (reverse). The β-actin primers were synthesized from Invitrogen (Shanghai, China) as follows: 5′-CCG TAA AGA CCT CTA TGC CAC AC-3′ (forward) and 5′-GGG TGT AAA ACG CAG CTC AGT A− 3′ (reverse). Real-time PCR reaction system was prepared according to the kit instructions, and the system was heated to 95°C for 30 seconds, followed by 40 cycles of heating up (95°C for 5 seconds) and cooling down (60°C for 30 seconds). Finally, the melting curve was collected. Real-time PCR amplification was performed using CFX96 Touch fluorescent quantitative PCR (Bio-Rad, Hercules, CA). Amplification of β-actin was used as an internal control.

### Cell Surface Staining

Cells were washed twice in PBS and blocked in PBS buffer containing 1% BSA for 30 min. Then, the cells were stained with conjugated antibodies that were specific for cell surface antigens for 30 min at 4°C in the dark. The stained immune cells were analysed by using flow cytometry (Beckman Coulter, Fullerton, CA), and the results were analysed with CytoExpert 2.3 software (Beckman Coulter).

### Intracellular Cytokine and Molecular Staining of Cells

Single-cell suspensions from the liver were stimulated with 20 ng/mL phorbol 12-myristate 13-acetate (PMA) plus 1 µg/mL ionomycin for 5 h at 37°C under a 5% CO_2_ atmosphere. Brefeldin A (10 g/mL, Sigma, Shanghai, China) was added during the last 4 h of incubation. Cells were washed twice in PBS, fixed with 4% paraformaldehyde, and permeabilized overnight at 4°C in PBS buffer containing 0.1% saponin (Sigma), 0.1% BSA, and 0.05% NaN_3_. Cells were then stained for 30 min at 4°C in the dark with conjugated antibodies specific for cell surface antigens as well as intracellular cytokines or proteins. The expression phenotypes of the antibody-labelled immune cells were analysed by flow cytometry (Beckman Coulter, Fullerton, CA), and the results were analysed with CytoExpert 2.3 software (Beckman Coulter). Isotype-matched cytokine controls were included in each staining protocol.

### SEA Preparation

SEA of *S. japonicum* cercariae was obtained from Jiangsu Institute of Parasitic Diseases (China). SEA was sterile filtered, and endotoxin was removed with the use of polymyxin B agarose beads (Sigma). The Limulus amoebocyte lysate assay kit (Lonza, Switzerland) was used to confirm the removal of endotoxins from the SEA.

### ELISA

The strips were coated with SEA overnight at 4°C and washed 3-5 times the next day. Then, the strips were covered with 10% FBS, 200 µL per well, for 1h. The plate was washed 3-5 times, and 100 µL of serum with the corresponding dilution ratio was added to each well and incubated at 37°C for 2h. The plate was washed 3-5 times, and HRP enzyme-labeled antibody was added at a corresponding dilution ratio of 100 µL in each well and incubated at 37°C for 1h. The plate was washed 5 times, 100 µL of substrate was added to each well and reacted for 5-30 mins, dilute sulfuric acid was added to terminate the reaction, and an enzyme label instrument was used to detect the results.

### Statistics

Statistical analysis between different groups was performed using unpaired t tests. The software packages GraphPad Prism version 5.0a and SPSS Statistics 17.0 were used. *P* < 0.05 was considered statistically significant.

## Results

### TLR7 KO Accelerates *S. japonicum* Infection-Induced Hepatitis

**Figure 1:**
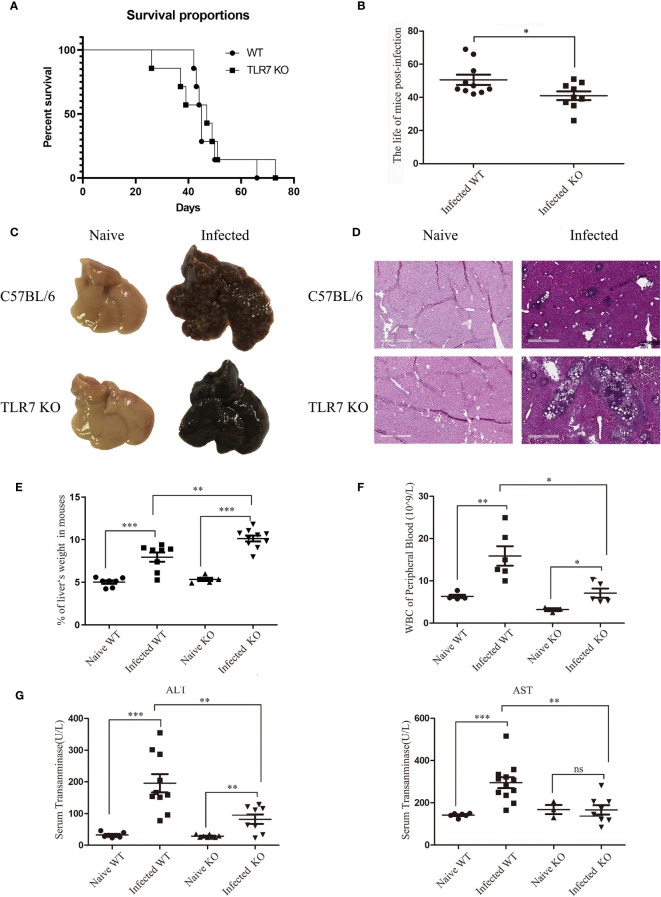
The pathological features of C57BL/6 mice during *S. japonicum infection*. Survival curve and characteristic pathological changes of mice infected with *S. japonicum*. WT and TLR7 KO mice were infected with *S. japonicum.* The survival times of naive and TLR7 KO mice after infection was recorded, and compared **(A, B)**. The dead mice were dissected, and gross changes in the liver in naive WT and TLR7 KO mice before and after infection were observed **(C)**. Haematoxylin and eosin staining was performed on paraffin sections of liver tissue from infected WT and TLR7 KO mice. Liver tissue sections from naive mice were used as controls **(D)**. The ratio of liver to body weight in WT and TLR7 KO mice before and after infection was calculated **(E)**. Peripheral white blood cells were counted **(F)**, and the levels of glutamic-pyruvic transaminase (ALT) and glutamic-oxalacetic transaminase (AST) in serum were detected **(G)**. Data were obtained from three independent experiments with 6-8 mice in each group, shown as the mean ± SEM. Statistical significance was determined by Student’s *t* test, “ns” means no sense, *P < 0.05, **P < 0.01, and ***P < 0.001.

To explore the role of TLR7 in mice infected with *S. japonicum*, the survival rates of wild-type (WT) and TLR7 knockout (KO) mice infected with *S. japonicum* were recorded. As shown in **Figure 1A**, 85.71% of WT mice died between 42 and 50 days, while the remaining WT mice died within 66 days. In contrast, 42.86% of TlR7 KO mice died within 40 days after infection, 42.86% of TLR7 KO mice died within 47-51 days, and 14.28% of TLR7 KO mice died within 73 days. However, TLR7 KO mice had a shorter survival time after infection than WT mice (**Figure 1B**). In summary, this evidence indicated that TLR7 KO mice were susceptible to death during the acute infection stage.

The mice that died naturally during infection were dissected, and the livers were weighed. The proportion of the liver weight in mice was approximately 10%, which was 2-fold that in naive mice(**Supplemental Figure 1**). Because the liver is the target organ during *S. japonicum* infection, the proportion of the liver in mice was used as an index of infection conditions. Two groups of *S. japonicum-*infected WT and TLR7 KO mice were sacrificed at 42 d postinfection. The proportion of the liver weight in WT mice was 7.94%, while it significantly increased to 10.13% in TLR7 KO mice, indicating that the condition of TLR7 KO mice was more serious than that of WT mice (**Figure 1E**).

To further investigate the difference between WT and TLR7 KO mice, HE sections of the liver were made 5-6 weeks after infection to analyse the pathological condition. Many white spots could be seen on the surface of WT livers, while the surface of TLR7 KO livers was smooth, with rare white spots (**Figure 1C**). A large number of granulomas surrounding the egg were observed in the liver tissue of infected WT mice, but few granulomas were found in infected TLR7 KO liver tissue, in which there were many nude eggs, indicating poor granuloma reaction in TLR7 KO mice (**Figure 1D**).

To further elucidate the roles of TLR7 in *S. japonicum* infection-induced hepatitis, blood was collected from both normal and infected WT and TLR7 KO mice, and serum aminotransferase (ALT and AST) and white blood cells (WBCs) were detected. The results indicated that the levels of WBC, ALT and AST increased significantly after infection (*P* < 0.05). Compared to the infected WT mice, the counts of WBCs in the blood and the levels of ALT and AST were lower in the serum of infected TLR7 KO mice (*P* < 0.05, **Figures 1F, G**). These results suggested that TLR7 enhanced *S. japonicum* infection-induced inflammation in the body.

### Cellular Distribution of TLR7 in the Livers of Infected Mice

**Figure 2:**
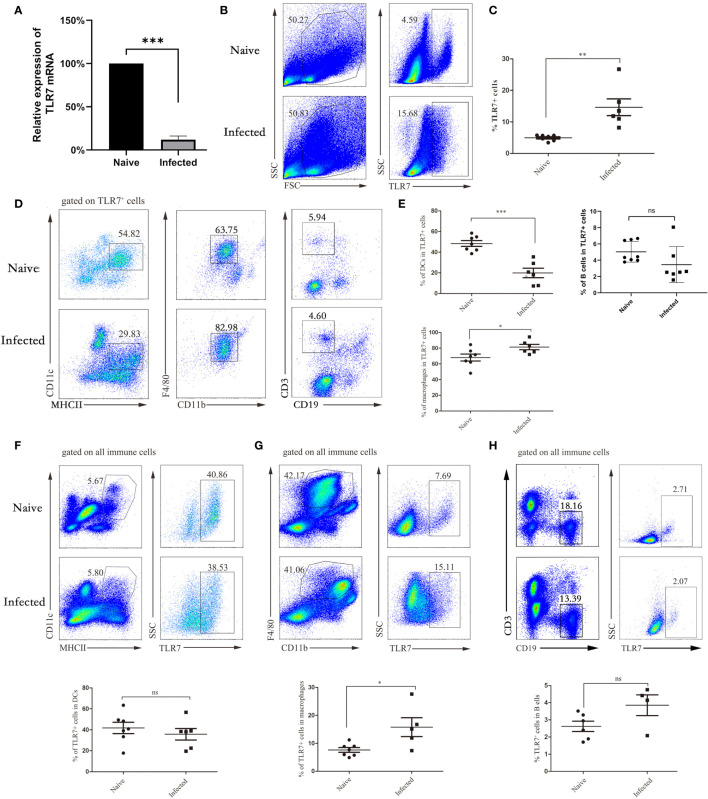
The distribution of TLR7 in APCs in the liver. WT mice were infected with *S. japonicum*, and livers were removed. Single-cell suspensions of liver were isolated from naive and infected mice. Total RNA of liver tissues from both naive and infected WT mice was extracted, cDNA was synthesized, and fluorescence quantitative PCR was performed to identify the expression of the TLR7 gene. The results are expressed as the percentage of gene expression relative to the same genotype **(A)**. Representative graphs and statistical analyses of the percentages of TLR7^+^ cells in the isolated lymphocytes were detected by FACS **(B, C)**. Representative graphs of DCs (CD11c^+^ MHCII^+^), macrophages (F4/80^+^ CD11b^+^) and B cells (CD3^-^CD19^+^) isolated from liver immune cells of WT and infected mice are shown **(D, E)**. The proportion of DCs to TLR7^+^ cells in normal and infected mice was statistically analysed, and the proportion of macrophages and B cells was also counted. The expression of TLR7 in DCs, macrophages and B cells was also analysed. Representative graphs and statistical analyses are shown **(F–H)**. Data were obtained from three independent experiments with 6-8 mice in each group, shown as the mean ± SEM. Statistical significance was determined by Student’s t test, “ns” means no sense, *P < 0.05, **P < 0.01, and ***P < 0.001.

To investigate the role of TLR7 during *S. japonicum infection*, we infected C57BL/6 mice with 40 ± 5 cercarias. After 5-6 weeks, the infected mice were sacrificed. Lymphocytes were isolated from the liver, and the expression of the TLR7 gene was identified by fluorescence quantitative PCR. As shown in **Figure 2A**, the expression of the TLR7 gene in lymphocytes from infected mice (88.06 ± 3.17%) was significantly decreased compared with that in lymphocytes from naive mice (*P* < 0.05). Furthermore, intracellular cytokine staining was performed to detect the proportion of TLR7-expressing lymphocytes in the liver. The results indicated that the proportion of TLR7-positive cells was markedly increased 5-6 weeks post infection (*P* < 0.05) and was more than 2-fold upregulated in infected mice (**Figures 2B, C**). This result suggested that TLR7 might modulate *S. japonicum* infection-induced hepatitis. Moreover, to explore the distribution of TLR7 in the isolated hepatic lymphocytes, TLR7-positive cells were gated first. The percentages of

MHCII^+^CD11C^+^ DCs, CD19^+^ B cells, and CD11b^+^F4/80^+^ macrophages were detected by FACS. As shown in **Figures 2D, E**, the percentage of MHCII^+^CD11C^+^ DC cells decreased significantly (*P* < 0.05), while the proportion of B cells did not change significantly, the percentage of CD11b^+^F4/80^+^ macrophages markedly increased in TLR7^+^ hepatic lymphocytes in the infected mice (*P* < 0.05), and most TLR7-expressing cells in the livers of infected mice were macrophages. This result indicated that TLR7 mainly established its role through macrophages in the livers of *S. japonicum-*infected mice. Moreover, MHCII^+^CD11C^+^ DCs, CD11b^+^F4/80^+^ macrophages and CD19^+^ B cells were gated first, and the expression of TLR7 was detected by FACs. As shown in **Figures 2F–H**, the percentage of TLR7^+^ cells in macrophages increased significantly, which was consistent with the above results.

### TLR7 Modulated the Responses of Hepatic Macrophages

**Figure 3:**
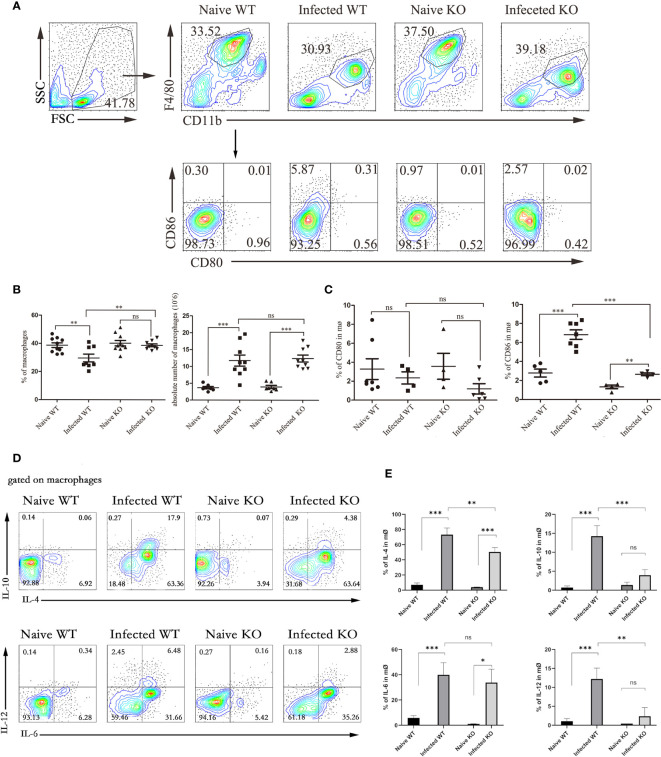
The function of liver macrophages during *S. japonicum infection*. WT and TLR7 KO mice were infected by *S. japonicum*. Five to six weeks later, livers were dissected out. Single-cell suspensions were separated from WT and KO mice before and after infection and stained with monoclonal antibodies against mouse F4/80, CD11b, CD80, and CD86. The expression of IL-4, IL-6, IL-10 and IL-12 was detected by intracellular cytokine staining. The strategy of gating macrophages is shown, and representative graphs of the expression of CD80 and CD86 in macrophages from WT and KO mice before and after infection are shown **(A)**. The percentage and absolute number of macrophages were compared between WT and KO mice before and after infection **(B)**. The expression of CD80 and CD80 was statistically analysed **(C)**. Gated on macrophages, the expression of IL-4, IL-6, IL-10 and IL-12 was detected. Representative graphs and statistical analyses are shown **(D, E)**. Data were obtained from three independent experiments with 6-8 mice in each group, shown as the mean ± SEM. Statistical significance was determined by Student’s t test, “ns” means no sense, *P < 0.05, **P < 0.01, and ***P < 0.001.

To further explore the role of TLR7 in modulating the immune response of macrophages in the livers of *S. japonicum*-infected mice, hepatic lymphocytes were isolated from both naive and infected WT and TLR7 KO mice 5-6 weeks after infection. The percentage of F4/80^+^CD11b^+^ macrophages was first compared. As shown in **Figures 3A, B**, the results indicated that although the percentage of macrophages was not increased significantly in the liver after *S. japonicum* infection (*P* > 0.05), the absolute numbers of macrophages in the liver markedly increased (*P* < 0.05). This result suggested that macrophages were involved in *S. japonicum* infection-induced hepatic inflammation. However, the percentage of macrophages in the livers of infected TLR7 KO mice was higher than that in the livers of infected WT mice (*P* < 0.05), and there was no significant difference in the absolute number of macrophages between infected WT and infected TLR7 KO mice (*P* > 0.05). This implied that TLR7 did not directly affect the expansion of hepatic macrophages.

In addition, the expression of costimulators CD80 and CD86 was detected on the surface of macrophages. As shown in **Figures 3A, C**, the results indicated that the expression of CD86 in infected mice significantly increased compared to that in naive mice (*P* < 0.05), and the expression of CD86 in infected TLR7 KO mice was significantly lower than that in infected WT mice (*P* < 0.05). However, the changes in the expression of CD80 were not significant, either before and after infection or between WT-infected and KO mice (*P* > 0.05).

Moreover, cells were stimulated by PMA and ionomycin, and the secretion of IL-6, IL-12, IL-10, and IL-4 was also detected by intracellular cytokine staining. As shown in **Figures 3D, E**, the results indicated that the secretion of these cytokines was increased significantly, especially IL-4, which increased from 10% to approximately 50% (*P* < 0.05). Compared with infected WT mice, infected KO mice exhibited significantly reduced secretion of these cytokines, except for IL-6 (*P* < 0.05).

### TLR7 Knockout Inhibited the Immune Response of Hepatic DCs

**Figure 4:**
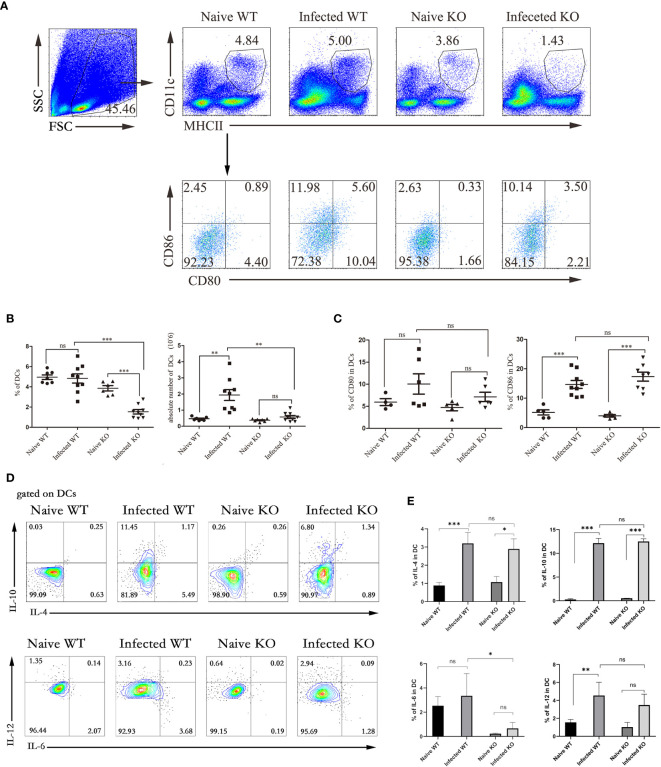
The function of liver DCs during *S. japonicum infection*. WT and TLR7 KO mice were infected by *S. japonicum*. Five to six weeks later, livers were dissected out. Single-cell suspensions were separated from WT and KO mice before and after infection and stained with monoclonal antibodies against mouse MHCII, CD11c, CD80, and CD86. The expression of IL-4, IL-6, IL-10 and IL-12 was detected by intracellular cytokine staining. The strategy of gating DCs is shown, and representative graphs of the expression of CD80 and CD86 in DCs from WT and KO mice before and after infection are shown **(A)**. The percentage and absolute number of DCs were compared between WT and KO mice before and after infection **(B)**. The expression of CD80 and CD80 was statistically analysed **(C)**. Gated on DCs, the expression of IL-4, IL-6, IL-10 and IL-12 was detected. Representative graphs and statistical analyses are shown **(D, E)**. Data were obtained from three independent experiments with 6-8 mice in each group, shown as the mean ± SEM. Statistical significance was determined by Student’s t test, “ns” means no sense, *P < 0.05, **P < 0.01, and ***P < 0.001.

DCs are potent APCs that activate naive T cells. The counts of MHCII^+^CD11C^+^ DCs in hepatic lymphocytes isolated from both naive and infected WT and TLR7 KO mice 5-6 weeks after infection were compared. As shown in **Figures 4A, B**, regardless of the proportion or absolute number, the DCs from infected TLR7 KO mice decreased significantly compared with those from infected WT mice (*P* < 0.05). This result indicated that hepatic DCs might play an important role in the course of *S. japonicum* infection. More interestingly, the proportion of DCs in the livers of infected TLR7 KO mice was only 1.43%, which was significantly lower than that in the other groups (3%-6%) (*P* < 0.05). There was no significant difference in the absolute number of DCs in KO mice before and after infection (*P* > 0.05). This implied that TLR7 might be involved in the expansion of DCs in the course of *S. japonicum* infection.

Although the CD86 in infected mice significantly increased compared to naive mice (*P* < 0.05), the expression of CD80 and CD86 was not significantly different between infected WT mice and infected TLR7 KO mice (**Figures 4A, C**). The results of cytokine secretion showed that although the percentages of IL-4-, IL-10-, and IL-12-secreting DCs from WT mice increased significantly after infection (*P* < 0.05), no marked difference was found between infected WT and infected TLR7 KO mice (*P* > 0.05, **Figures 4D, E**).

### TLR7 Enhances the Th2 Immune Response in the Course of *S. japonicum* Infection

**Figure 5:**
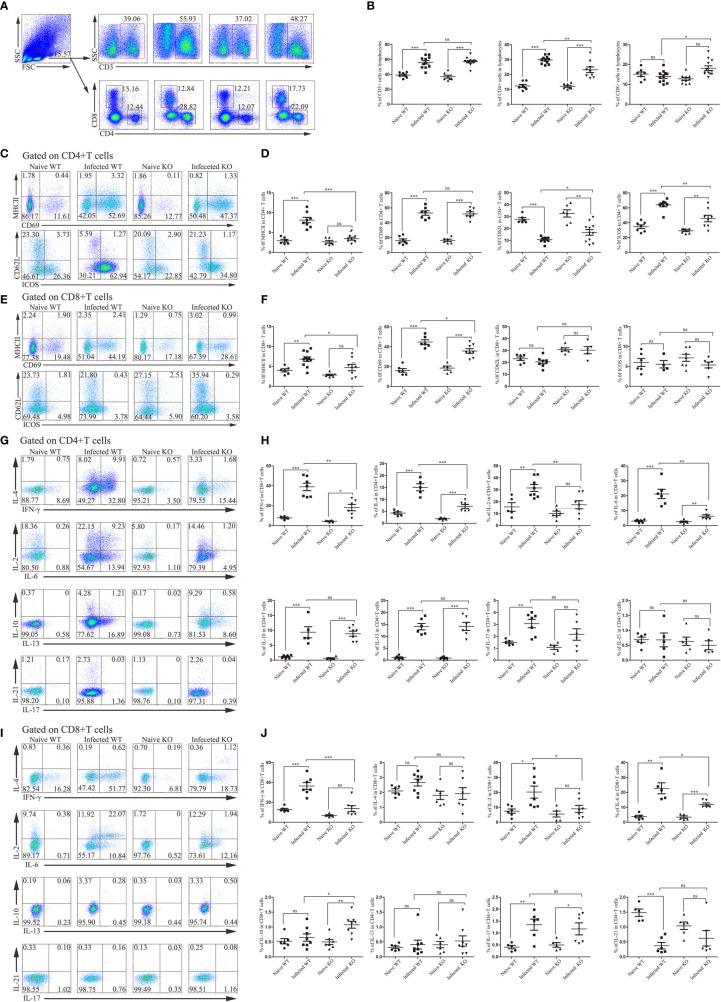
The activation and function of liver CD4^+^ T lymphocytes and CD8^+^ T lymphocytes during *S. japonicum* infection. WT and TLR7 KO mice were infected by *S. japonicum*. Five to six weeks later, livers were dissected out. Single-cell suspensions were separated from WT and KO mice before and after infection and stained with monoclonal antibodies against mouse CD3, CD4, CD8, MHCII, CD69, CD62L and ICOS. Meanwhile, under stimulation with PMA plus ionomycin, the expression of IL-2, IL-4, IL-6, IFN-γ, IL-10, IL-13, IL-17 and IL-21 was detected by intracellular cytokine staining. Representative graphs and the proportions of CD3^+^, CD4^+^ and CD8^+^ T cells are shown **(A, B)**. Then, CD4^+^ and CD8^+^ T cells were gated. The expression of MHCII, CD69, CD62L and ICOS was examined. Representative graphs and statistical analyses of CD4^+^ and CD8^+^ T cells in WT and KO mice before and after infection are shown **(C–F)**. The expression of the abovementioned intracellular cytokines was also detected by flow cytometry. Representative graphs and statistical analyses of CD4^+^ and CD8^+^ T cells in both naive and infected WT and KO mice **(G–J)**. Data were obtained from three independent experiments with 6-8 mice in each group, shown as the mean ± SEM. Statistical significance was determined by Student’s t test, “ns” means no sense, *P < 0.05, **P < 0.01, and ***P < 0.001.

The next step is to investigate whether these changes depend on the effects on T lymphocytes, surface markers and secretory cytokines of T lymphocytes. After *S*. *japonicum* infection, liver T lymphocytes, mainly CD4^+^ T cells, increased (*P* < 0.05). Compared with infected WT mice, the total T lymphocytes of infected KO mice did not change significantly, but the decrease in CD4^+^ T cells was obvious (*P* < 0.05). Interestingly, the proportion of CD8^+^ T cells increased slightly (*P* < 0.05, **Figures 5A, B**). These results suggested that deletion of TLR7 might reduce the recruitment of CD4^+^ T cells.

CD4^+^ T cells and CD8^+^ T cells from both WT and TLR7 KO mice expressed higher levels of activation-associated molecules after infection for 6 weeks (*P* < 0.05). The percentage of activated CD4^+^ T cells in TLR7 KO mice was significantly decreased compared to that in infected WT mice (*P* < 0.05). Only MHC II and CD69 were significantly decreased in CD8^+^ T cells from infected TLR7 KO mice compared to infected WT mice (**Figures 5C–F**).

Furthermore, intracellular cytokine staining was performed on hepatic CD4^+^ T cells and CD8^+^ T cells isolated from different groups of mice. As shown in **Figures 5I, J**, most of the detected cytokines secreted by CD4^+^ T cells from infected WT mice were increased significantly compared to naive mice, such as IFN-γ, IL-4, IL-2 and IL-6 (*P* < 0.05). IFN-γ, IL-4, IL-2 and IL-6 secreted by CD4^+^ T cells from infected TLR7 KO mice significantly decreased compared to infected WT mice (*P* < 0.05, **Figures 5G, H**). A similar phenomenon has been observed in CD8^+^ T cells. The percentage of IFN-γ-secreting CD8^+^ T cells increased significantly after infection (*P* < 0.05), while it decreased significantly in infected TLR7 KO mice, as well as IL-2 and IL-6 (*P7* < 0.05).

### TLR7 Enhanced the Hepatic B Cell Response in the Course of *S. japonicum* Infection

**Figure 6:**
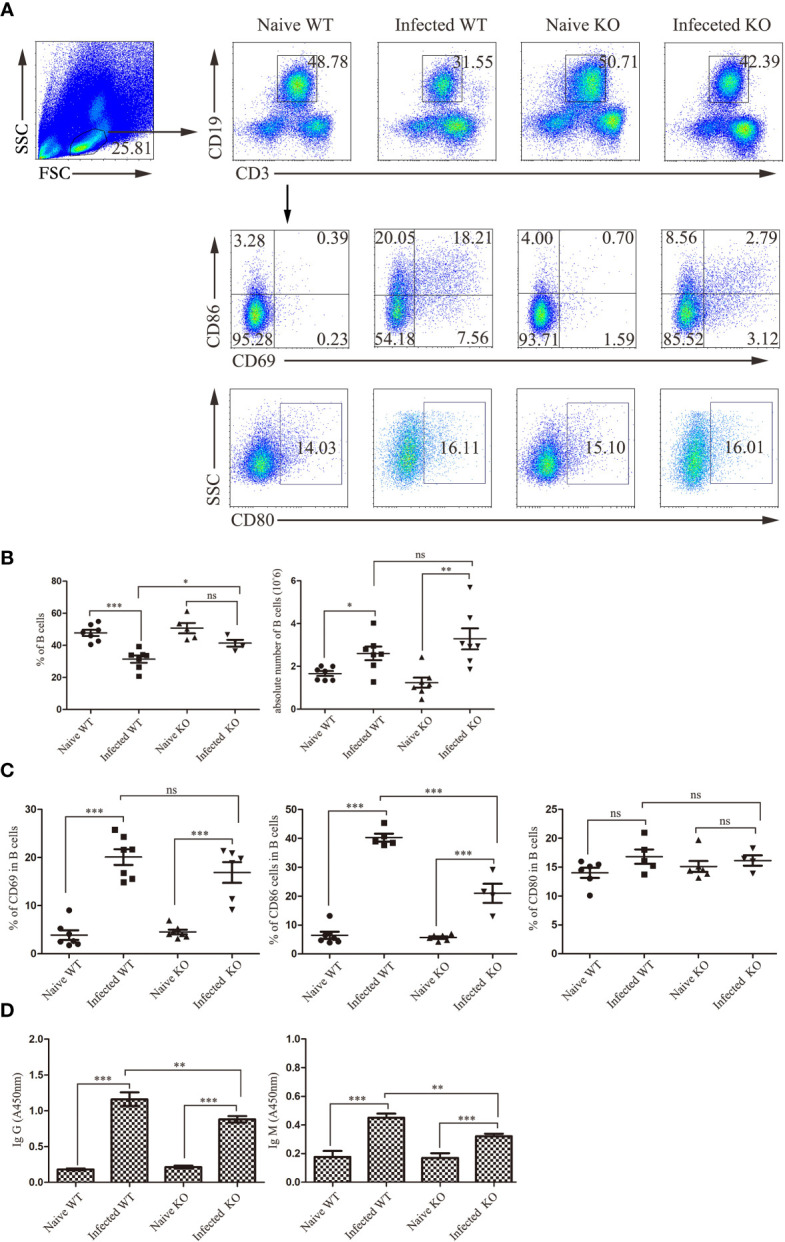
The activation and function of liver B lymphocytes during *S. japonicum* infection. WT and TLR7 KO mice were infected by *S. japonicum*. Five to six weeks later, livers were picked out. Single-cell suspensions were separated from WT and KO mice before and after infection and stained with monoclonal antibodies against mouse CD69, CD80 and CD86. The strategy of gating B cells is shown, and representative graphs of the expression of CD69, CD80 and CD86 in B cells from WT and KO mice before and after infection are shown **(A)**. The percentage and absolute number of B cells were compared between WT and KO mice before and after infection **(B)**. The expression of CD69, CD80 and CD80 was statistically analysed **(C)**. The SEA-specific antibody in mouse serum was detected by ELISA. The relative titres of anti-SEA IgG and anti-SEA IgM in the serum of WT and KO mice before and after infection were statistically analysed **(D)**. Data were obtained from three independent experiments with 6-8 mice in each group, shown as the mean ± SEM. Statistical significance was determined by Student’s t test, “ns” means no sense, *P < 0.05, **P < 0.01, and ***P < 0.001.

In the course of *S. japonicum* infection, B cells play an important role. The role of TLR7 in modulating the B cell response in the course of *S. japonicum* infection was also explored. As shown in **Figures 6A, B**, compared to naive mice, the proportion of CD19^+^ B cells in infected mice was decreased significantly (*P* < 0.05). The absolute numbers of CD19^+^ B cells increased significantly (*P* < 0.05). There was no change between uninfected and infected KO mice in the percentage of CD19^+^ B cells (*P >* 0.05).

Furthermore, activation-associated markers were detected on hepatic B cells from different groups of mice. As shown in **Figures 6A, C**, there was no difference in the proportion of B cells expressing CD69 and CD80 between WT- and KO-infected mice (*P >* 0.05), with only a significant decrease in the proportion of CD86 in the latter (*P* < 0.05).

In addition, SEA-specific antibodies were detected in the serum of mice (**Figure 6D**). The levels of SEA-specific IgM and IgG antibodies in infected mice were significantly higher than those in naive mice (*P <* 0.05). Moreover, the levels of SEA-specific IgM and IgG antibodies in the serum of infected TLR7 KO mice were markedly decreased compared to those in infected WT mice (*P* < 0.05). These results suggested that TLR7 might play an important role in inducing antibody production in the course of *S. japonicum* infection.

## Discussion

TLR7 is an endosomal TLR that recognizes single-stranded RNA (ssRNA) and mediates the development of inflammation and autoimmunity (Luo et al., 2019; Souyris et al., 2019). In this study, both C57BL/6 mice (WT) and TLR7 KO mice were infected with *S. japonicum.* The survival results showed that the majority of infected mice did not die before 6 weeks post infection, while approximately half of TLR7 KO mice died within 6 weeks post infection, indicating that TLR7 may protect the host from death in the early phase of *S*. *japonicum* infection. *S. japonicum* ovulates eggs at approximately 4 weeks post infection, some of which deposit in the liver and cause granulomas to form around them (Pagan and Ramakrishnan, 2018). Once schistosomiasis progresses into the advanced phase, the host will always die from cirrhosis of the liver caused by granuloma inflammation (Malta et al., 2021). Reduced granulomatous formation did not improve host status and resulted in significant mortality, indicating that granulomatous inflammation can lead to pathological changes; on the other hand, granulomatous inflammation limits the spread of infection and plays a role in host protection (Hams et al., 2013). It is hypothesized that the death of TLR7 KO mice in the early phase of *S. japonicum* infection may be related to the absence of granulomas around the eggs. Therefore, TLR7 KO mice that died within 6 weeks post infection were dissected, and the livers were sectioned. There was poor granuloma formation in the liver of the host, and the liver tissue was filled with eggs of *S. japonicum*, which suggests that the deletion of TLR7 reduces the formation of granulomas during infection.

Next, the expression of TLR7 in hepatic lymphocytes was investigated. The results indicated that the relative TLR7 mRNA level in hepatic lymphocytes from infected mice was lower than that in hepatic lymphocytes from naive mice. This might be induced by the amount of infection, recruiting lower TLR7-expressing lymphocytes, such as T cells and B cells, as we observed in mesenteric lymph nodes (Qu et al., 2019). This hypertension was validated by the FCM results, which indicated that the percentage of TLR7-expressing cells was increased in the isolated hepatic lymphocytes. Macrophages, DCs, and B cells are classic APCs that express many kinds of TLRs, including TLR7, and modulate the adaptive immune response (Assier et al., 2007). FCM results showed that in the isolated hepatic lymphocytes, most TLR7-expressing cells were CD11b^+^F4/80^+^ macrophages. On the other hand, the percentage of TLR7^+^ macrophages increased significantly in the livers of *S*. *japonicum*-infected mice. These results implied that TLR7 might modulate the *S*. *japonicum* infection-induced immune response mainly through macrophages. It was reported that activated macrophages (AAM) induced early in the anti-helminth response could amplify the early type 2 immune cascade initiated by epithelial cells and ILC2s, and subsequently driving parasite expulsion (Coakley and Harris, 2020). It suggested TLR7 triggering responses in liver resident macrophages mainly influenced the subsequent immune response in the liver of *S*. *japonicum* infected mouse.

Liver-resident macrophages, also called Kupffer cells (KCs), are professional APCs that can modulate the class T cell response by providing costimulators and producing cytokines (Fu et al., 2020). CD80 and CD86 are important costimulators for T cell activation (Trzupek et al., 2020), and IL-12 and IL-4 are cytokines that induce Th2 and Th1 polarization, respectively (Cui et al., 2017). IL-6 and IL-10 are important proinflammatory cytokines and anti-inflammatory cytokines, respectively (Kumar et al., 2021). Higher numbers of macrophages were found in the livers of infected WT and TLR7 KO mice, which expressed higher levels of CD86 and secreted more IL-4, IL-10, and IL-12, suggesting that macrophages played an important role in the progression of *S*. *japonicum* infection-induced hepatitis, as previously reported (Tan et al., 2019; Ye et al., 2020). Although the number of macrophages in the livers of infected TLR7 KO mice was similar to that in infected WT mice, they expressed lower CD86 levels and produced fewer cytokines. This result suggested that TLR7 is a main molecule that mediates the function of hepatic macrophages in the course of *S*. *japonicum* infection.

Dendritic cells are the only antigen-presenting cells that can activate naive T lymphocytes. It was reported that incomplete deletion of dendritic cells severely impairs the induction and development of the Th2 response (Phythian-Adams et al., 2010). A significant increase in the number of DCs in infected mouse livers, with higher CD86 expression and IL-4, IL-10 and IL-12 secretion, suggested that DCs were involved in *S. japonicum* infection-induced hepatitis. Moreover, the percentage and number of DCs in TLR7 KO mice at 6 weeks post infection significantly decreased compared to WT mice, and the percentage of IL-6-secreting DCs also decreased. This result suggested that the deletion of TLR7 significantly impaired the function of hepatic DCs, which could effect on the subsequent immune response in the liver of infected mouse, too.

The formation of granulomas in *S. japonicum*-infected mice depends on the Th2 response, and the deletion of T cells impaired granuloma formation around eggs (Kumar et al., 2019). It was reported that TLR7 could enhance the type 2 immune response in many kinds of diseases (Chodisetti et al., 2020). In this study, the number and percentage of CD4^+^ T cells and the percentage of IL-4^+^CD4^+^ Th cells in hepatic lymphocytes from infected TLR7 KO mice decreased significantly compared to those from infected WT mice. This result suggested that TLR7 plays an important role in enhancing the Th2 response during *S. japonicum* infection. Strangely, the secretion of IFN-γ in TLR7 KO mice also significantly decreased compared to that in infected WT mice, which means that TLR7 modulates not only the Th2 response but also the Th1 response. Consistent with our results, administration of a TLR7 agonist was reported to induce a mixed Th1 and Th2 response in peripheral blood mononuclear cells in chickens (Annamalai et al., 2015).

In addition to differentiating into plasma cells to secrete antibodies, B cells are professional APCs that play important roles in the presentation of soluble antigens (Leon et al., 2019). Recently, reports showed that a subset of B lymphocytes can also activate naive T lymphocytes (Hong et al., 2018; Arroyo and Pepper, 2020). Higher numbers of B cells were found in the livers of infected WT mice expressing higher levels of CD69 and CD86, suggesting that hepatic B cells could mediate the T cell response in *S. japonicum* infection-induced hepatitis. In addition, these results implied that hepatic B cells could modulate the response of Th cells in turn. Recently, TLR7 was reported to be expressed on B cells and mediate the subsequent immune response (Zheng et al., 2020; Fillatreau et al., 2021). In this study, a higher percentage of B cells was found in TLR7-expressing hepatic lymphocytes, suggesting that TLR7 might directly mediate the immune response of B cells in the livers of infected mice. In addition, CD4^+^ Th2 cells are the main source of cells responsible for B cell activation and differentiation (Xiao et al., 2020). As mentioned before, TLR7 could enhance the response of Th2 cells in the livers of infected mice, and TLR7 could affect the responses of B cells through Th2 cells. Moreover, it was reported that APCs could help B cell activation *via* the formation of an immune synapse (Wang et al., 2018). This implied that APCs might be another pathway by which TLR7 regulates the *S. japonicum* infection-induced B cell response.

Altogether, this study indicated that TLR7 could delay the progression of *S. japonicum* infection-induced hepatitis by facilitating the formation of granulomas mainly through hepatic macrophages. DCs, B cells, and T cells are involved in TLR7-mediated immune responses.

## Data Availability Statement

The raw data supporting the conclusions of this article will be made available by the authors, without undue reservation.

## Ethics Statement

The animal study was reviewed and approved by Experimental Animal Ethics Committee of Guangzhou Medical University.

## Author Contributions

XP, XT, and JH conceived the study. YF, HX, and FS performed the *in vitro* cellular test. DC, AX, and JL performed histological experiment. CF and HW analysed the results. SY, HH, and HX prepared parasite and animal. JM, XW, and JH contributed to the writing of the paper. All authors contributed to the article and approved the submitted version.

## Funding

This research was supported by grants from the Natural Science Foundation of China (81771696, 81802024), the Natural Science Foundation of Guangdong province (2020A1515010251, 2021A1515011032), Guangzhou science and technology project (202002030082, Joint Project of Science and Technology Bureau and University from Guangzhou Municipal Science and Technology Bureau (No. 202102010130), and Special Clinical Technology of Guangzhou (No. TS36).

## Conflict of Interest

The authors declare that the research was conducted in the absence of any commercial or financial relationships that could be construed as a potential conflict of interest.

## Publisher’s Note

All claims expressed in this article are solely those of the authors and do not necessarily represent those of their affiliated organizations, or those of the publisher, the editors and the reviewers. Any product that may be evaluated in this article, or claim that may be made by its manufacturer, is not guaranteed or endorsed by the publisher.

## References

[B1] Annamalai A. Ramakrishnan S. Sachan S. Sharma B. K. Anand K. B. Kumar V.. (2015). Administration of TLR7 Agonist, Resiquimod, in Different Types of Chicken Induces a Mixed Th1 and Th2 Response in the Peripheral Blood Mononuclear Cells. Res. Vet. Sci. 100, 105–108. doi: 10.1016/j.rvsc.2015.04.007 25935758

[B2] Arroyo E. N. Pepper M. (2020). B Cells are Sufficient to Prime the Dominant CD4+ Tfh Response to Plasmodium Infection. J. Exp. Med. 217 (2), e20190849. doi: 10.1084/jem.20190849 31748243 PMC7041722

[B3] Assier E. Marin-Esteban V. Haziot A. Maggi E. Charron D. Mooney N. (2007). TLR7/8 Agonists Impair Monocyte-Derived Dendritic Cell Differentiation and Maturation. J. Leukoc. Biol. 81, 221–228. doi: 10.1189/jlb.0705385 17023556

[B4] Baccarella A. Fontana M. F. Chen E. C. Kim C. C. (2013). Toll-Like Receptor 7 Mediates Early Innate Immune Responses to Malaria. Infect. Immun. 81, 4431–4442. doi: 10.1128/IAI.00923-13 24042114 PMC3837989

[B5] Boni C. Vecchi A. Rossi M. Laccabue D. Giuberti T. Alfieri A.. (2018). TLR7 Agonist Increases Responses of Hepatitis B Virus-Specific T Cells and Natural Killer Cells in Patients With Chronic Hepatitis B Treated With Nucleos(T)Ide Analogues. Gastroenterology 154, 1764–1777. doi: 10.1053/j.gastro.2018.01.030 29378197

[B6] Cha H. Xie H. Jin C. Feng Y. Xie S. Xie A.. (2020). Adjustments of Gammadelta T Cells in the Lung of Schistosoma Japonicum-Infected C56BL/6 Mice. Front. Immunol. 11, 1045. doi: 10.3389/fimmu.2020.01045 32582168 PMC7287124

[B7] Chodisetti S. B. Fike A. J. Domeier P. P. Singh H. Choi N. M. Corradetti C.. (2020). Type II But Not Type I IFN Signaling Is Indispensable for TLR7-Promoted Development of Autoreactive B Cells and Systemic Autoimmunity. J. Immunol. 204, 796–809. doi: 10.4049/jimmunol.1901175 31900342 PMC7002260

[B8] Coakley G. Harris N. L. (2020). Interactions Between Macrophages and Helminths. Parasite Immunol. 42, e12717. doi: 10.1111/pim.12717 32249432

[B9] Cui A. H. Zhao J. Liu S. X. Hao Y. S. (2017). Associations of IL-4, IL-6, and IL-12 Levels in Peripheral Blood With Lung Function, Cellular Immune Function, and Quality of Life in Children With Moderate-to-Severe Asthma. Med. (Baltimore) 96, e6265. doi: 10.1097/MD.0000000000006265 PMC5371444 28328807

[B10] De Marco V. C. Potriquet J. You H. McManus D. P. Mulvenna J. Jones M. K. (2019). Qualitative and Quantitative Proteomic Analyses of Schistosoma Japonicum Eggs and Egg-Derived Secretory-Excretory Proteins. Parasit Vectors 12, 173. doi: 10.1186/s13071-019-3403-1 30992086 PMC6469072

[B11] Ernst O. Failayev H. Athamna M. He H. Tsfadia Y. Zor T. (2020). A Dual and Conflicting Role for Imiquimod in Inflammation: A TLR7 Agonist and a Camp Phosphodiesterase Inhibitor. Biochem. Pharmacol. 182, 114206. doi: 10.1016/j.bcp.2020.114206 32828805

[B12] Farwa A. He C. Xia L. Zhou H. (2018). Immune Modulation of Th1, Th2, and T-Reg Transcriptional Factors Differing From Cytokine Levels in Schistosoma Japonicum Infection. Parasitol Res. 117, 115–126. doi: 10.1007/s00436-017-5678-5 29188369

[B13] Fillatreau S. Manfroi B. Dorner T. (2021). Toll-Like Receptor Signalling in B Cells During Systemic Lupus Erythematosus. Nat. Rev. Rheumatol 17, 98–108. doi: 10.1038/s41584-020-00544-4 33339987 PMC7747191

[B14] Fu H. Y. Bao W. M. Yang C. X. Lai W. J. Xu J. M. Yu H. Y.. (2020). Kupffer Cells Regulate Natural Killer Cells via the NK Group 2, Member D (NKG2D)/Retinoic Acid Early Inducible-1 (RAE-1) Interaction and Cytokines in a Primary Biliary Cholangitis Mouse Model. Med. Sci. Monit 26, e923726. doi: 10.12659/MSM.923726 32599603 PMC7346879

[B15] Hams E. Aviello G. Fallon P. G. (2013). The Schistosoma Granuloma: Friend or Foe? Front. Immunol. 4, 89. doi: 10.3389/fimmu.2013.00089 23596444 PMC3625856

[B16] Heni A. C. Schmid J. Rasche A. Corman V. M. Drosten C. Sommer S. (2020). Pathogen-Associated Selection on Innate Immunity Genes (TLR4, TLR7) in a Neotropical Rodent in Landscapes Differing in Anthropogenic Disturbance. Heredity (Edinb) 125, 184–199. doi: 10.1038/s41437-020-0331-y 32616896 PMC7490709

[B17] Hong S. Zhang Z. Liu H. Tian M. Zhu X. Zhang Z.. (2018). B Cells Are the Dominant Antigen-Presenting Cells That Activate Naive CD4(+) T Cells Upon Immunization With a Virus-Derived Nanoparticle Antigen. Immunity 49, 695–708. doi: 10.1016/j.immuni.2018.08.012 30291027

[B18] Hu Y. Tang L. Zhu Z. Meng H. Chen T. Zhao S.. (2020). A Novel TLR7 Agonist as Adjuvant to Stimulate High Quality Hbsag-Specific Immune Responses in an HBV Mouse Model. J. Transl. Med. 18, 112. doi: 10.1186/s12967-020-02275-2 32131853 PMC7055022

[B19] Huwait E. A. Al-Ghamdi M. A. Ghattas M. H. Hinnis A. R. El-Maaty D. Abo-Elmatty D. M.. (2021). Role of Heme Oxygenase-1, Cytokines, and Vascular Endothelial Growth Factor in Murine Schistosoma Mansoni. Int. J. Health Sci. (Qassim) 15, 22–28. 33456439 PMC7786444

[B20] Karadimou G. Folkersen L. Berg M. Perisic L. Discacciati A. Roy J.. (2017). Low TLR7 Gene Expression in Atherosclerotic Plaques Is Associated With Major Adverse Cardio- and Cerebrovascular Events. Cardiovasc. Res. 113, 30–39. doi: 10.1093/cvr/cvw231 27864310 PMC5220676

[B21] Kassa B. Mickael C. Kumar R. Sanders L. Koyanagi D. Hernandez-Saavedra D.. (2019). Paclitaxel Blocks Th2-Mediated TGF-Beta Activation in Schistosoma Mansoni-Induced Pulmonary Hypertension. Pulm. Circ. 9, 767726899. doi: 10.1177/2045894018820813 PMC6304706 30511588

[B22] Knolle P. A. Wohlleber D. (2016). Immunological Functions of Liver Sinusoidal Endothelial Cells. Cell Mol. Immunol. 13, 347–353. doi: 10.1038/cmi.2016.5 27041636 PMC4856811

[B23] Kumar R. Mickael C. Kassa B. Sanders L. Koyanagi D. Hernandez-Saavedra D.. (2019). Th2 CD4(+) T Cells Are Necessary and Sufficient for Schistosoma-Pulmonary Hypertension. J. Am. Heart Assoc. 8, e13111. doi: 10.1161/JAHA.119.013111 PMC6761627 31339057

[B24] Kumar A. Sharma S. P. Agarwal A. Gupta V. Katoch D. Sehgal S.. (2021). Tear IL-6 and IL-10 Levels in HLA-B27-Associated Uveitis and Its Clinical Implications. Ocul. Immunol. Inflammation 29, 237–243. doi: 10.1080/09273948.2019.1704022 31940227

[B25] Lackey E. K. Horrall S. (2020). Schistosomiasis (Treasure Island (FL): StatPearls Publishing). 32119321

[B26] Leon M. A. Wemlinger S. M. Larson N. R. Ruffalo J. K. Sestak J. O. Middaugh C. R.. (2019). Soluble Antigen Arrays for Selective Desensitization of Insulin-Reactive B Cells. Mol. Pharm. 16, 1563–1572. doi: 10.1021/acs.molpharmaceut.8b01250 30681867 PMC7446942

[B27] Llanwarne F. Helmby H. (2021). Granuloma Formation and Tissue Pathology in Schistosoma Japonicum Versus Schistosoma Mansoni Infections. Parasite Immunol. 43, e12778. doi: 10.1111/pim.12778 32692855 PMC11478942

[B28] Luo Z. Su R. Wang W. Liang Y. Zeng X. Shereen M. A.. (2019). EV71 Infection Induces Neurodegeneration *via* Activating TLR7 Signaling and IL-6 Production. PloS Pathog. 15, e1008142. doi: 10.1371/journal.ppat.1008142 31730654 PMC6932824

[B29] Malta K. K. Silva T. P. Palazzi C. Neves V. H. Carmo L. Cardoso S. J.. (2021). Changing Our View of the Schistosoma Granuloma to an Ecological Standpoint. Biol. Rev. Camb Philos. Soc. 96 (4), 1404–1420. doi: 10.1111/brv.12708 33754464

[B30] Michaelis K. A. Norgard M. A. Zhu X. Levasseur P. R. Sivagnanam S. Liudahl S. M.. (2019). The TLR7/8 Agonist R848 Remodels Tumor and Host Responses to Promote Survival in Pancreatic Cancer. Nat. Commun. 10, 4682. doi: 10.1038/s41467-019-12657-w 31615993 PMC6794326

[B31] Osada Y. Horie Y. Nakae S. Sudo K. Kanazawa T. (2019). STAT6 and IL-10 Are Required for the Anti-Arthritic Effects of Schistosoma Mansoni *via* Different Mechanisms. Clin. Exp. Immunol. 195, 109–120. doi: 10.1111/cei.13214 30194773 PMC6300695

[B32] Pagan A. J. Ramakrishnan L. (2018). The Formation and Function of Granulomas. Annu. Rev. Immunol. 36, 639–665. doi: 10.1146/annurev-immunol-032712-100022 29400999

[B33] Parra F. L. Caimi A. T. Altube M. J. Cargnelutti D. E. Vermeulen M. E. de Farias M. A.. (2018). Make It Simple: (SR-A1+TLR7) Macrophage Targeted Nanoarchaeosomes. Front. Bioeng. Biotechnol. 6, 163. doi: 10.3389/fbioe.2018.00163 30460231 PMC6232313

[B34] Paveley R. A. Aynsley S. A. Turner J. D. Bourke C. D. Jenkins S. J. Cook P. C.. (2011). The Mannose Receptor (CD206) Is an Important Pattern Recognition Receptor (PRR) in the Detection of the Infective Stage of the Helminth Schistosoma Mansoni and Modulates Ifngamma Production. Int. J. Parasitol 41, 1335–1345. doi: 10.1016/j.ijpara.2011.08.005 22036898

[B35] Phythian-Adams A. T. Cook P. C. Lundie R. J. Jones L. H. Smith K. A. Barr T. A.. (2010). CD11c Depletion Severely Disrupts Th2 Induction and Development *In Vivo* . J. Exp. Med. 207, 2089–2096. doi: 10.1084/jem.20100734 20819926 PMC2947067

[B36] Qu J. Yu X. Jin C. Feng Y. Xie S. Xie H.. (2019). TLR7 Modulated T Cell Response in the Mesenteric Lymph Node of Schistosoma Japonicum-Infected C57BL/6 Mice. J. Immunol. Res. 2019, 2691808. doi: 10.1155/2019/2691808 31930147 PMC6942828

[B37] Racanelli V. Rehermann B. (2006). The Liver as an Immunological Organ. Hepatology 43, S54–S62. doi: 10.1002/hep.21060 16447271

[B38] Regli I. B. Passelli K. Martinez-Salazar B. Amore J. Hurrell B. P. Muller A. J.. (2020). TLR7 Sensing by Neutrophils Is Critical for the Control of Cutaneous Leishmaniasis. Cell Rep. 31, 107746. doi: 10.1016/j.celrep.2020.107746 32521271

[B39] Saroa R. Kaushik D. Bagai U. Kaur S. Salunke D. B. (2019). Efficacy of TLR7 Agonistic Imidazoquinoline as Immunochemotherapeutic Agent Against P. Berghei ANKA Infected Rodent Host. Bioorg Med. Chem. Lett. 29, 1099–1105. doi: 10.1016/j.bmcl.2019.02.029 30850167

[B40] Schwartz C. Fallon P. G. (2018). Schistosoma “Eggs-Iting” the Host: Granuloma Formation and Egg Excretion. Front. Immunol. 9, 2492. doi: 10.3389/fimmu.2018.02492 30459767 PMC6232930

[B41] Souyris M. Mejia J. E. Chaumeil J. Guery J. C. (2019). Female Predisposition to TLR7-Driven Autoimmunity: Gene Dosage and the Escape From X Chromosome Inactivation. Semin. Immunopathol. 41, 153–164. doi: 10.1007/s00281-018-0712-y 30276444

[B42] Tang C. L. Gao Y. R. Wang L. X. Zhu Y. W. Pan Q. Zhang R. H.. (2019). Role of Regulatory T Cells in Schistosoma-Mediated Protection Against Type 1 Diabetes. Mol. Cell Endocrinol. 491, 110434. doi: 10.1016/j.mce.2019.04.014 31078638

[B43] Tan Z. Lei Z. Zhang Z. Zhang H. Shu K. Hu F.. (2019). Identification and Characterization of Microglia/Macrophages in the Granuloma Microenvironment of Encephalic Schistosomiasis Japonicum. BMC Infect. Dis. 19, 1088. doi: 10.1186/s12879-019-4725-5 31888505 PMC6937796

[B44] Trzupek D. Dunstan M. Cutler A. J. Lee M. Godfrey L. Jarvis L.. (2020). Discovery of CD80 and CD86 as Recent Activation Markers on Regulatory T Cells by Protein-RNA Single-Cell Analysis. Genome Med. 12, 55. doi: 10.1186/s13073-020-00756-z 32580776 PMC7315544

[B45] Wang J. C. Bolger-Munro M. Gold M. R. (2018). Imaging the Interactions Between B Cells and Antigen-Presenting Cells. Methods Mol. Biol. 1707, 131–161. doi: 10.1007/978-1-4939-7474-0_10 29388105

[B46] Wang X. Li L. Wang J. Dong L. Shu Y. Liang Y.. (2017). Inhibition of Cytokine Response to TLR Stimulation and Alleviation of Collagen-Induced Arthritis in Mice by Schistosoma Japonicum Peptide SJMHE1. J. Cell Mol. Med. 21, 475–486. doi: 10.1111/jcmm.12991 27677654 PMC5323857

[B47] Wang X. Y. Xu J. Zhao S. Li W. Zhang J. F. He J.. (2018). Estimating the Prevalence of Schistosomiasis Japonica in China: A Serological Approach. Infect. Dis. Poverty 7, 62. doi: 10.1186/s40249-018-0443-2 29961423 PMC6027568

[B48] Williamson T. Sultanpuram N. Sendi H. (2019). The Role of Liver Microenvironment in Hepatic Metastasis. Clin. Transl. Med. 8, 21. doi: 10.1186/s40169-019-0237-6 31263976 PMC6603103

[B49] Wohlleber D. Knolle P. A. (2016). The Role of Liver Sinusoidal Cells in Local Hepatic Immune Surveillance. Clin. Transl. Immunol. 5, e117. doi: 10.1038/cti.2016.74 PMC5192065 28090319

[B50] Xiao J. Guan F. Sun L. Zhang Y. Zhang X. Lu S.. (2020). B Cells Induced by Schistosoma Japonicum Infection Display Diverse Regulatory Phenotypes and Modulate CD4(+) T Cell Response. Parasit Vectors 13, 147. doi: 10.1186/s13071-020-04015-3 32197642 PMC7082913

[B51] Ye Z. Huang S. Zhang Y. Mei X. Zheng H. Li M.. (2020). Galectins, Eosinophiles, and Macrophages may Contribute to Schistosoma Japonicum Egg-Induced Immunopathology in a Mouse Model. Front. Immunol. 11, 146. doi: 10.3389/fimmu.2020.00146 32231658 PMC7082360

[B52] Zheng N. Xie K. Ye H. Dong Y. Wang B. Luo N.. (2020). TLR7 in B Cells Promotes Renal Inflammation and Gd-Iga1 Synthesis in Iga Nephropathy. JCI Insight 5 (14), e136965. doi: 10.1172/jci.insight.136965 PMC7453916 32699192

