# Supplementary material for: Roles of TLR7 in Schistosoma japonicum Infection-Induced Hepatic Pathological Changes in C57BL/6 Mice
Source: Front Cell Infect Microbiol. 2021 Oct 8;11:754299. doi: 10.3389/fcimb.2021.754299 (PMC8531751; doi:10.3389/fcimb.2021.754299)
Supplement: Supplementary file 2 [file Table_2.docx]

**Supplemental Information**


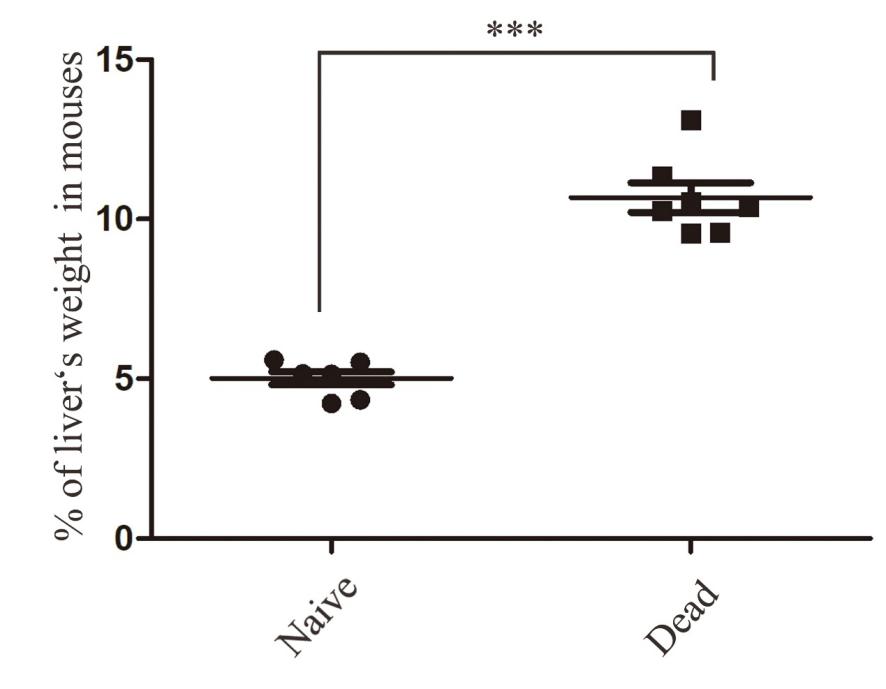


**Figure S1: Comparison of liver weight ratio between normal mice and infected dead mice**

Mice were infected with *S. japonicum* or not. After the infected mice was dead, the body was weighted, and the liver was picked out, and weighted, too. The rate of liver,s weight in mouse was counted. The difference between naive and infected mouse was compared. ***, *P*<0.001.
